# Association Between Anthropometric Risk Factors and Metabolic Syndrome Among Adults in India: A Systematic Review and Meta-Analysis of Observational Studies

**DOI:** 10.5888/pcd19.210231

**Published:** 2022-05-05

**Authors:** Yuvaraj Krishnamoorthy, Sathish Rajaa, Sharan Murali, Jayaprakash Sahoo, Sitanshu Sekhar Kar

**Affiliations:** 1 Department of Community Medicine, Employee State Insurance Corporation Medical College and Post Graduate Institute of Medical Science & Research, K.K. Nagar, Chennai, India; 2 Senior Resident, Department of Preventive and Social Medicine, Jawaharlal Institute of Postgraduate Medical Education and Research, Puducherry, India; 3 Department of Endocrinology, Jawaharlal Institute of Postgraduate Medical Education and Research, Puducherry, India

## Abstract

**Introduction:**

Several studies have explored the effect of anthropometric risk factors on metabolic syndrome. However, no systematic effort has explored the effect of overweight and obesity on the prevalence of metabolic syndrome in India. Thus, we undertook a meta-analysis to estimate the effect of anthropometric risk factors on the prevalence of metabolic syndrome.

**Methods:**

We searched databases PubMed Central, EMBASE, MEDLINE, and Cochrane library and search engines ScienceDirect and Google Scholar, from January 1964 through March 2021. We used the Newcastle–Ottawa scale to assess the quality of published studies, conducted a meta-analysis with a random-effects model, and reported pooled odds ratios (OR) with 95% CIs.

**Results:**

We analyzed 26 studies with a total of 37,965 participants. Most studies had good to satisfactory quality on the Newcastle–Ottawa scale. Participants who were overweight (pooled OR, 5.47; 95% CI, 3.70–8.09) or obese (pooled OR, 5.00; 95% CI, 3.61–6.93) had higher odds of having metabolic syndrome than those of normal or low body weight. Sensitivity analysis showed no significant variation in the magnitude or direction of outcome, indicating the lack of influence of a single study on the overall pooled estimate.

**Conclusion:**

Overweight and obesity are significantly associated with metabolic syndrome. On the basis of evidence, clinicians and policy makers should implement weight reduction strategies among patients and the general population.

**Summary**

**What is already known on this topic?**

Researchers have tested various treatment options to reduce the prevalence of metabolic syndrome, such as lifestyle and diet modifications, pharmacologic therapy, weight reduction, behavioral therapy, and bariatric surgery.

**What is added by this report?**

Overweight and obese adults have about 5 times higher odds of having metabolic syndrome than adults with normal or low body weight.

**What are the implications for public health practice?**

Given the evidence linking obesity to metabolic syndrome, clinicians and policy makers should recommend weight management measures to patients and the general population. Interventions promoting physical activity should be developed and implemented in phases in high-prevalence settings.

## Introduction

Metabolic syndrome encompasses a spectrum of disorders, including central obesity, atherogenic dyslipidemia (ie, low high-density lipoprotein cholesterol [HDL-C], elevated triglycerides, and apolipoprotein B–containing lipoproteins), elevated blood pressure, elevated blood glucose, and prothrombotic and proinflammatory states (1). Metabolic syndrome recently emerged as a significant and growing public health challenge worldwide resulting from rapid urbanization, excessive energy intake, developing obesity, and sedentary lifestyle habits (2). People with metabolic syndrome have increased risk of type 2 diabetes mellitus, cardiovascular disease, myocardial infarction, and stroke and twice the risk of death from these causes compared with people without the syndrome (3).

Metabolic syndrome is characterized by chronic low-grade inflammation, which is caused by complex interactions between genetic and environmental factors (4). Prevalence has varied from 10% to 84% worldwide, depending on both the criteria used for diagnosis (5) and differences in the geographic distribution, ethnicity, age, and sex of the population studied (6). A recent meta-analysis showed that the prevalence of metabolic syndrome in India is 30% and is more commonly seen among older adults (>60 y), women, and the urban population (7). However, research exploring factors that determine this high prevalence is limited. Factors such as genetic susceptibility, obesity, physical inactivity, smoking, and alcohol consumption are components of the syndrome's natural history (8).

Several studies have explored the reasons why obesity and physical inactivity affect metabolic syndrome. Adipocyte hypertrophy and hyperplasia, enhanced by obesity and overweight, influence the overproduction of biologically active metabolites, known as adipocytokines, such as free fatty acids, tumor necrosis factor-α, interleukin-6, and plasminogen activator inhibitor-1 (9). These mediators initiate a localized and systemic inflammation that facilitates multiple processes, such as insulin insensitivity, oxidant stress, blood coagulation, and inflammatory responses, that in turn accelerate atherosclerosis (10). Researchers have experimented with various treatment options, such as lifestyle and diet modifications, pharmacologic therapy, weight reduction, behavioral therapy, and bariatric surgery, to reduce the syndrome’s prevalence (2).

Existing evidence on anthropometric factors related to metabolic syndrome is not country-specific; however, it is essential to know whether the influence of these factors differs from country to country. Although India has almost one-third of the world’s adult population with metabolic syndrome, no systematic effort has been made to explore the effect of overweight and obesity on the syndrome’s prevalence in India. To develop effective strategies and implement relevant policies or programs to address the prevalence of metabolic syndrome, policy makers must have information on its contributing factors. However, we found no systematic review to date that examined the association worldwide between anthropometric factors and metabolic syndrome. Hence, we undertook our meta-analysis to estimate the effect of anthropometric risk factors on the prevalence of metabolic syndrome to inform researchers in India and worldwide.

## Methods

### Design and registration

We conducted a systematic review and meta-analysis of observational studies. The protocol was registered in PROSPERO under the registration number CRD42019147277. The “Preferred Reporting Items for Systematic Reviews and Meta-Analyses (PRISMA) statement 2020” was used to report this systematic review incorporating the meta-analyses (11). The institutional review board of Jawaharlal Institute of Postgraduate Medical Education and Research declared this study exempt from review.

### Eligibility criteria

We included any observational study irrespective of its design (ie, cross-sectional, case-control, cohort) that reported the relevant exposure (anthropometric risk factors) and outcome (metabolic syndrome) in India. We did not restrict studies by geographic region (rural or urban) or study setting (community, facility, workplace). Only full-text publications were included, and we excluded studies published as conference abstracts, case reports, or case series and unpublished data.

The studies included were conducted among adults in India aged 18 years or older and assessed the association of anthropometric risk factors (overweight/obesity) with metabolic syndrome. We excluded disease-specific (eg, noncommunicable diseases, mental health disorders) studies. Studies reporting prevalence of metabolic syndrome in relation to different anthropometric factors were included irrespective of the type of definition or criteria used for diagnosis (eg, National Cholesterol Education Program Adult Treatment Panel III guidelines, International Diabetes Federation guidelines, Harmonized Asia Pacific criteria).

We conducted a systematic search of literature in electronic databases (PubMed Central, EMBASE, MEDLINE, and Cochrane library) and by using search engines (ScienceDirect and Google Scholar). Both medical subject headings (MeSH) and free-text words were used for all searches (Table 1). We used a set of keywords and their synonyms for searches with appropriate truncations and wildcards and for proximity searching. We also searched for key concepts by using corresponding subject headings in each of the databases. Our final search was conducted by combining individual search results by using appropriate Boolean operators (“OR” and “AND”). The search was narrowed by using the available filters for time period (from inception of the databases [January 1964 through March 2021]), language (published in English language only), and study design (observational studies). Bibliographies of the retrieved articles were also hand-searched to identify any articles missed during the database search.

**Table 1:** Search Strategy, Association Between Anthropometric Risk Factors and Metabolic Syndrome Among Adults in India

Search engine	Search terms	Search results
EMBASE	('india'/exp OR 'india' OR 'indian union' OR 'republic of india' OR 'union of india') AND ('adult'/exp OR 'adult' OR 'adults' OR 'grown-ups' OR 'grownup' OR 'grownups') AND ('physical activity'/exp OR 'activity, physical' OR 'physical activity' OR 'alcohol'/exp OR 'ablysinol' OR 'alcohol' OR 'alcohol concentration' OR 'alcohol vapor' OR 'alcohol vapour' OR 'alcohol, ethyl' OR 'dehydrated alcohol' OR 'dehydrated ethanol' OR 'dehydrated ethyl alcohol' OR 'ethanol' OR 'ethanol solution' OR 'ethyl alcohol' OR 'ethylalcohol' OR 'obesity'/exp OR 'adipose tissue hyperplasia' OR 'adipositas' OR 'adiposity' OR 'alimentary obesity' OR 'body weight, excess' OR 'corpulency' OR 'fat overload syndrome' OR 'nutritional obesity' OR 'obesitas' OR 'obesity' OR 'overweight' OR 'smoking'/exp OR 'behavior, smoking' OR 'behaviour, smoking' OR 'reverse smoking' OR 'smoker' OR 'smokers' OR 'smoking' OR 'smoking behavior' OR 'smoking behaviour' OR 'tobacco smoking') AND ('metabolic syndrome x'/exp OR 'insulin resistance syndrome' OR 'metabolic syndrome' OR 'metabolic syndrome x' OR 'syndrome x, metabolic') AND ('article'/it OR 'article in press'/it)	1,891 Results
PubMed	("physical activity"[Title/Abstract] OR "physical inactivity"[Title/Abstract] OR "exercise"[Title/Abstract] OR "physical exercise"[Title/Abstract] OR "smoking"[Title/Abstract] OR "smoker"[Title/Abstract] OR "alcohol"[Title/Abstract] OR "alcoholic"[Title/Abstract] OR "overweight"[Title/Abstract] OR "high bmi"[Title/Abstract] OR "obesity"[Title/Abstract] OR "obese"[Title/Abstract]) AND "metabolic syndrome"[Title/Abstract] AND ("india"[MeSH Terms] OR "india"[All Fields] OR "india s"[All Fields] OR "indias"[All Fields])) AND (english[Filter]) Translations india: "india"[MeSH Terms] OR "india"[All Fields] OR "india's"[All Fields] OR "indias"[All Fields]	823 Results

### Study selection

Our study selection process involved 3 stages:

1. Primary screening: Two independent investigators (Y.K. and S.R.) performed primary screening of title, abstract, and keywords by executing the literature search. Full-text articles were retrieved for the studies shortlisted on the basis of eligibility criteria.
2. Secondary screening: Full text of these retrieved studies was screened by Y.K. and S.R. and assessed against our eligibility criteria. We included studies that satisfied all eligibility criteria with respect to design, participants, exposure, and outcome.
3. Finalizing the study selection: Disagreements among investigators during the screening process were resolved, and final consensus on inclusion of studies was reached with the help of another investigator (S.M.). Where the required information was missing, we contacted the corresponding author of the respective study and obtained the information. If we received no response from the author, the study was excluded.

### Data extraction

We manually extracted data by using a predefined, structured data extraction form that included general information about the article, such as author and year of publication, and information related to the methods section, including study design, setting, sample size, sampling strategy, study participants, inclusion and exclusion criteria, exposure and outcome assessment method, quality-related information, the number of participants in exposed and nonexposed groups, and the number of exposed and nonexposed participants with metabolic syndrome. Data were entered by S.R., and entries were double-checked by Y.K. and S.M. for accuracy. We based our criteria for a diagnosis of metabolic syndrome on those of the National Cholesterol Education Program Adult Treatment Panel III (NCEP ATP III) (4), the International Diabetes Federation (3), the American Heart Association and the National Heart, Lung, and Blood Institute (4), and the Harmonized Asia Pacific criteria (4) **(** Box **)**.

Box. Criteria for Diagnosis of Metabolic Syndrome, by Organization **National Cholesterol Education Program Adult Treatment Panel III (4)**

Three or more of the following:

Waist circumference: ≥102 cm in men; ≥88 cm in women
Triglyceride: ≥1.7 mmol/L or treatment for elevated trigylceride
High-density lipoprotein cholesterol (HDL-C): men, <1.03 mmol/L; women, <1.29 mmol/L; or undergoing treatment for low HDL-C
Blood pressure: systolic ≥130 mmHg or diastolic ≥85 mm Hg or hypertension treatment or previously diagnosed hypertension
Fasting blood glucose: ≥5.6 mmol/L, treatment for elevated blood glucose, or previously diagnosed type 2 diabetes

**International Diabetes Federation (3)**

Increased waist circumference (men, ≥90 cm; women,  ≥80 cm) plus 2 or more of the following:

Triglyceride: ≥1.7 mmol/L or treatment for elevated triglyceride
HDL-C: men,  <1.03 mmol/L; women,  <1.29 mmol/L; or treatment for low HDL-C
Blood pressure: systolic,  ≥130 mm Hg; diastolic,  ≥85 mm Hg; hypertension treatment; or previously diagnosed hypertension
Fasting blood glucose: ≥5.6 mmol/L, treatment for elevated blood glucose, or previously diagnosed type 2 diabetes

**American Heart Association and the National Heart, Lung, and Blood Institute (4):**

Three or more of the following:

Waist circumference: men, ≥40 inches (≥102 cm); women, ≥35 inches (≥89 cm), measured at the top of the iliac crest at the end of a normal expiration
Triglyceride: ≥150 mg per dL (≥1.70 mmol per L) or receiving pharmacologic therapy for elevated triglyceride levels
HDL-C: men, <40 mg/per dL (<1.05 mmol per L); women, <50 mg per dL (<1.30 mmol per L); or receiving pharmacologic therapy for low HDL-C
Blood pressure: systolic, ≥130 mm Hg; diastolic, ≥85 mm Hg, or receiving pharmacologic therapy for hypertension
Fasting blood glucose: ≥100 mg per dL (≥5.6 mmol per L) or receiving pharmacologic therapy for elevated fasting blood glucose level

**Harmonized Asia Pacific (4)**

Three or more of the following 5 risk factors:

Increased waist circumference: men, ≥90 cm; women, ≥80 cm
Triglyceride level of  ≥1.7 mmol/L
HDL-C: men,  <1.0 mmol/L; women,  <1.3 mmol/L
Blood pressure: systolic, ≥130 mm Hg; diastolic, ≥ 85 mm Hg; or current use of antihypertensive medications
Fasting blood sugar:  ≥5.6 mmol/L or currently using antidiabetes medications

### Risk-of-bias assessment

Two independent investigators, S.R. and S.M., used the Newcastle–Ottawa Quality Assessment Scale to perform risk-of-bias assessment for observational studies (12). The scale consists of 3 domains, each of which receives a score of stars that varies by category: selection (maximum 4 stars), comparability (maximum 2 stars), and outcome (maximum 2 stars). Within each of these domains, we assessed representativeness, sample size justification, nonresponse, ascertainment of exposure, control for confounding, assessment of outcome, and statistical tests. The total score for a study ranged from 0 to 8 stars. Studies with 7 to 8 stars were considered of good quality; 5 to 6, of satisfactory quality; and 0 to 4, of unsatisfactory quality (12).

### Statistical analysis

We used STATA version 14.2 (StataCorp) to perform our meta-analysis. Because all outcomes were dichotomous, the number of events and participants in each group were entered to obtain the pooled effect estimate in terms of odds ratio (OR). We used the random effects model with the inverse variance method to calculate the weight of individual studies. Evidence of between-study variance resulting from heterogeneity was assessed through a χ^2^ test of heterogeneity and by using *I* ^2^ statistics to quantify the inconsistency. An *I* ^2^ less than 25% indicates mild heterogeneity; 25%–75%, moderate; and more than 75%, substantial (13). Study-specific and pooled estimates were graphically represented through forest plots. We also performed subgroup analysis by using multiple study characteristics or covariates, study setting, geographic region, metabolic syndrome definitions, and quality of studies. We were able to perform analysis based on study design because only 1 study was a prospective study; the rest were cross-sectional.

We performed univariable meta-regression analysis with study-level characteristics. Variables with a *P* value less than .20 were used to perform multivariable meta-regression for identifying the source of heterogeneity between the studies. We assessed publication bias for each of the outcomes by using a funnel plot and a Doi plot (14) for visual interpretation and Egger test (13) and the Luis Furuya-Kanamori asymmetry index (LFK index) (14) for statistical interpretation. Asymmetry of the funnel plot or the Doi plot and a *P* value less than .10 in the Egger test indicates possible publication bias. On the basis of the LFK index value, the possibility of publication bias is classified as no asymmetry (value within ±1), minor asymmetry (value greater than ±1 but within ±2), and major asymmetry (value greater than ±2) (14).

We performed sensitivity analysis to assess the robustness of results by removing the studies one by one and checking for any significant variation in results. We also performed random-effects cumulative meta-analysis to delineate temporal changes in the magnitude and direction of the pooled association estimate because the evidence accumulates over time. First, we sorted the studies by publication year and then added them sequentially to analysis in chronological order, recalculating the pooled estimates with each added study (15).

## Results

### Study selection

**Figure 1:**
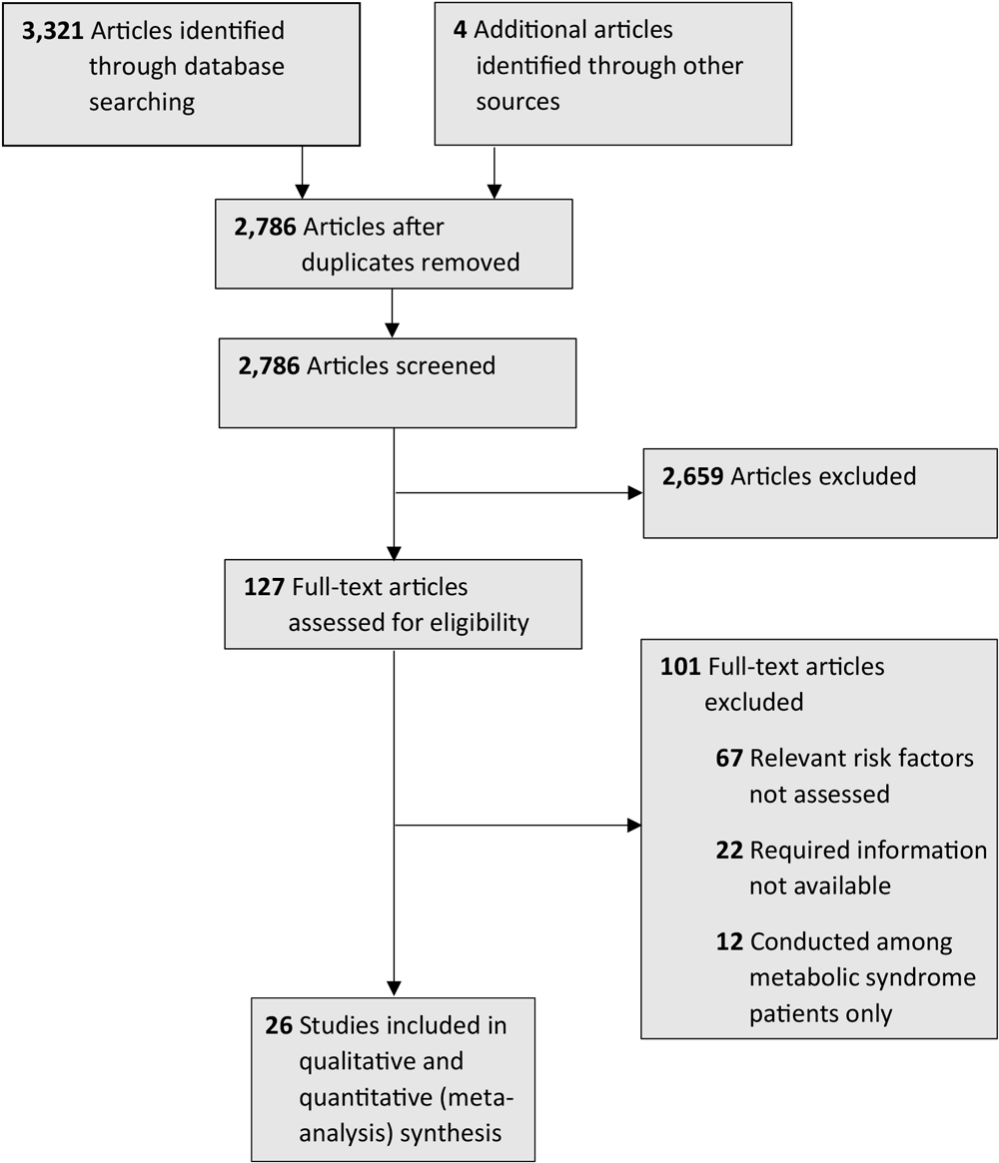
Flowchart describing the selection process for the 26 studies (16–41) included in the systematic review of studies on the association between anthropometric risk factors and metabolic syndrome among adults in India.

We found 3,321 studies through our systematic literature search. We also retrieved full texts for 4 articles obtained through manual searching of the bibliographies in the retrieved studies. After removing duplicates, we screened 2,786 articles. Of these, we excluded 2,659 because the title and abstract indicated that they did not have relevant study participants or exposure or outcomes. We assessed 127 for eligibility and excluded 101 (67 because relevant risk factors weren’t assessed, 22 because required information was not available, and 12 because the studies described were conducted among metabolic syndrome patients only). A total of 26 studies with a total of 37,965 participants were included in our final review for qualitative and quantitative (meta-analysis) synthesis and met our eligibility criteria (16–41). We used a PRISMA (Preferred Reporting Items for Systematic Reviews and Meta-Analyses) flow chart to describe the screening and selection process (Figure 1).

Half of the included studies (13 of 26) were conducted in the southern states of Tamil Nadu, Kerala, Andhra Pradesh, and Telangana (18,22–24,28,29,31,34,36–40). Nineteen were community-based studies (16,17,19,21–26,28–34,36,38,41), 6 were facility-based (18,20,27,35,37,40), and 1 (39) was a workplace-based study. The sample sizes of the included studies ranged from 112 to 9,886 participants. Many (9 of 26) were conducted in an urban region (17,19,21,28,29,31,33,36,38). Six were conducted in a rural region (16,24,25,32,34,41) and 1 in a tribal region (23). The average age of study participants ranged from 19.6 to 69.5 years. Most studies (17 of 26) used NCEP ATP-III criteria to diagnose metabolic syndrome (16,18,21–24,26,27,30–32,34,35,37–39,41). Twenty-one (17–23,25–28,30–35,37,38,40,41) reported an association between metabolic syndrome and obesity, and 15 (16–19,24,27–29,31,32,35–37,39,40) found the syndrome to be associated with overweight (Table 2).

**Table 2:** Characteristics of Studies (N = 26) of Anthropometric Risk Factors and Metabolic Syndrome Among Adults, January 1964–March 2021

Author, year, type^a^	State, region, type	Setting, participant sex^b^	Age, y	Sample size	Criteria for exposure (criteria for metabolic syndrome^c^)
Barik et al, 2018 (16)	West Bengal, East, rural	Community	≥18	9,886	NCEP ATP III (overweight, BMI ≥23)
Bhagat et al, 2017 (17)	Chandigarh, North, urban	Community	18–25	611	IDF (overweight, BMI ≥23, obesity; BMI ≥25)
Bhattacharya et al, 2016 (18)	Telangana, South, NA	Facility	≥60	114	NCEP ATP -III (overweight, BMI ≥23; obesity, BMI ≥27.5)
Deedwania et al, 2014 (19)	11 cities in India, urban	Community	≥20	6,198	Asia-Pacific criteria (overweight, BMI ≥23; obesity, BMI ≥25)
Goyal et al, 2014 (20)	Uttarakhand, North, NA	Facility	≥18	380	IDF (obesity, BMI ≥25)
Gupta et al, 2004 (21)	Rajasthan, North, urban	Community	≥20	1,071	NCEP ATP III (obesity, BMI ≥25)
Harikrishnan et al, 2018 (22)	Kerala, South, urban and rural	Community	≥20	5,063	NCEP ATP III (obesity, BMI ≥25)
Ismail et al, 2016 (23)	Kerala, South, tribal	Community	≥18	120	NCEP ATP III (obesity, BMI ≥25)
Jamkhandi et al, 2019 (24), prospective	Tamil Nadu, South, rural	Community, women only	38–45	200	NCEP ATP III (overweight, BMI ≥23)
Kapil et al, 2018 (25)	Uttarkhand, North, rural	Community	≥60	979	IDF (obesity, BMI ≥25)
Kaur et al, 2014 (26)	Punjab, North, urban and rural	Community	≥20	351	NCEP ATP III (obesity, BMI ≥25)
Kunti et al, 2019 (27)	West Bengal, East, NA	Facility	≥18	330	NCEP ATP III (overweight, BMI ≥23; obesity, BMI ≥25)
Lakshmipriya et al, 2013 (28)	Tamil Nadu, South, urban	Community	≥20	1,875	IDF (overweight, BMI ≥23; obesity, BMI ≥25)
Majumdar et al, 2017 (29)	Andhra Pradesh, South, urban	Community	≥60	112	IDF (overweight, BMI ≥23)
Mangat et al, 2010 (30)	Chandigarh, North, urban and rural	Community	≥18	605	NCEP ATP III (obesity, BMI ≥25)
Manjunath et al, 2014 (31)	Andhra Pradesh, South, urban	Community	18–25	473	NCEP ATP III (obesity, BMI ≥25)
Misra et al, 2011 (32)	Haryana, North, rural	Community	≥20	307	NCEP ATP III (overweight, BMI ≥23; obesity, BMI ≥25)
Muddegowda et al, 2016 (37)	Kerala, South, NA	Facility	≥20	432	NCEP ATP III (overweight, BMI ≥23; obesity, BMI ≥25)
Prasad et al, 2012 (33)	Orissa, East, urban	Community	20–80	1,178	IDF (obesity, BMI ≥25)
Selvaraj et al, 2019 (34)	Tamil Nadu, South, rural	Community, men only	20–40	360	NCEP ATP III (obesity, BMI ≥25)
Sharma et al, 2016 (35)	Chandigarh, North, NA	Facility, women only	45–55	350	NCEP ATP III (overweight, BMI ≥23; obesity, BMI ≥25)
Sinha et al, 2016 (36)	Telangana, South, urban	Community	≥60	114	IDF (overweight, BMI ≥23)
Tharkar et al, 2010 (38)	Tamil Nadu, South, urban	Community	≥20	2,021	NCEP ATP III (obesity, BMI ≥25)
Thayyil et al, 2012 (39)	Kerala, South, NA	Workplace, men only	≥30	823	NCEP ATP III (overweight, BMI ≥23)
Vembu et al, 2020 (40)	Tamil Nadu, South, NA	Facility, women only	19–40	1,030	AHA/NHLBI (overweight, BMI ≥23; obesity, BMI ≥25)
Zafar et al, 2017 (41)	Uttar Pradesh, West, rural	Community	18–55	2,982	NCEP ATP III (obesity, BMI ≥25)

Abbreviations: AHA/NHLBI, American Heart Association/National Heart, Lung, and Blood Institute; BMI, body mass index; IDF, International Diabetes Foundation; NA, not applicable; NCEP ATP III, National Cholesterol Education Program Adult Treatment Panel III.

a Studies were cross-sectional unless otherwise indicated.

b Studies included both men and women unless otherwise indicated.

c Physical activity, smoking, alcohol, overweight, obesity.

### Quality assessment

Under the selection bias domain, most studies (17 of 26) had high bias risk related to representativeness of the sample (17,18,20–22,25,26,29,31–37,39,40), about 15 had high bias risk related to sample size justification (18,20,21,26,28,29,33–41), and 16 had high bias risk related to nonresponse (16–22,25,28,31,33,34,38–41). However, none of the studies had high risk related to ascertainment of exposure. Under the comparability domain, 16 studies had high risk of bias related to control of confounding (17–21,23,26,29–32,34,35,37,40,41). Under the outcome domain, only 1 study had high bias risk related to ascertainment of outcome and statistical test reporting (40). More than half of the studies (14 of 26) had good to satisfactory quality (16,19,22–25,27,28,30,32,33,36,38,39) (Table 3).

**Table 3:** Bias Risk, by Domains of Quality Assessment^a^, Studies (N = 26) of Anthropometric Risk Factors and Metabolic Syndrome Among Adults, January 1964–March 2021

Author, year	Selection				Comparability	Outcome		
	Representativeness	Sample size justification	Non-response	Ascertainment of exposure	Control for confounding	Assessment of outcome	Statistical tests	Overall quality
Barik et al, 2018 (16)	1	1	0	1	2	1	1	Good
Bhagat et al, 2017 (17)	0	1	0	1	0	1	1	Unsatisfactory
Bhattacharya et al, 2016 (18)	0	0	0	1	0	1	0	Unsatisfactory
Deedwania et al, 2014 (19)	1	1	0	1	0	1	1	Satisfactory
Goyal et al, 2014 (20)	0	0	0	1	0	1	1	Unsatisfactory
Gupta et al, 2004 (21)	0	0	0	1	0	1	1	Unsatisfactory
Harikrishnan et al, 2018 (22)	0	1	0	1	2	1	1	Satisfactory
Ismail et al, 2016 (23)	1	1	1	1	0	1	1	Satisfactory
Jamkhandi et al, 2019 (24), prospective	1	1	1	1	2	1	1	Good
Kapil et al, 2018 (25)	0	1	0	1	2	1	1	Satisfactory
Kaur et al, 2014 (26)	0	0	1	1	0	1	1	Unsatisfactory
Kunti et al, 2019 (27)	1	1	1	1	2	1	1	Good
Lakshmipriya et al, 2013 (28)	1	0	0	1	2	1	1	Satisfactory
Majumdar et al, 2017 (29)	0	0	1	1	0	1	1	Unsatisfactory
Mangat et al, 2010 (30)	1	1	1	1	0	1	1	Satisfactory
Manjunath et al, 2014 (31)	0	1	0	1	0	1	1	Unsatisfactory
Misra et al, 2011 (32)	0	1	1	1	0	1	1	Satisfactory
Muddegowda et al, 2016 (37)	0	0	1	1	0	1	1	Unsatisfactory
Prasad et al, 2012 (33)	0	0	0	1	2	1	1	Satisfactory
Selvaraj et al, 2019 (34)	0	0	0	1	0	1	1	Unsatisfactory
Sharma et al, 2016 (35)	0	0	1	1	0	1	1	Unsatisfactory
Sinha et al, 2016 (36)	0	0	1	1	2	1	1	Satisfactory
Tharkar et al, 2010 (38)	1	0	0	1	2	1	1	Satisfactory
Thayyil et al, 2012 (39)	0	0	0	1	2	1	1	Satisfactory
Vembu et al, 2020 (40)	0	0	0	1	0	0	1	Unsatisfactory
Zafar et al, 2017 (41)	1	0	0	1	0	1	1	Unsatisfactory

a Calculated by using the Newcastle–Ottawa Quality Assessment Scale (NOS) (12). The scale consists of 3 domains, each of which receives a score of a number of stars that varies by category: selection (maximum 4 stars), comparability (maximum 2 stars), and outcome domains (maximum 2 stars). Under these domains, we assessed the following criteria: representativeness, sample size justification, nonresponse, ascertainment of exposure, control for confounding, assessment of outcome, and statistical tests. The total score for a study ranged from 0 to 8 stars. Studies with 7 to 8 stars are considered of good quality; 5 to 6, of satisfactory quality; and 0 to 4, of unsatisfactory quality. Values refer to the number of stars the study received. The higher the number, the higher the NOS rating. Zero indicates that the study was not evaluated in that category.

### Association of anthropometric risk factors with metabolic syndrome

**Figure 2:**
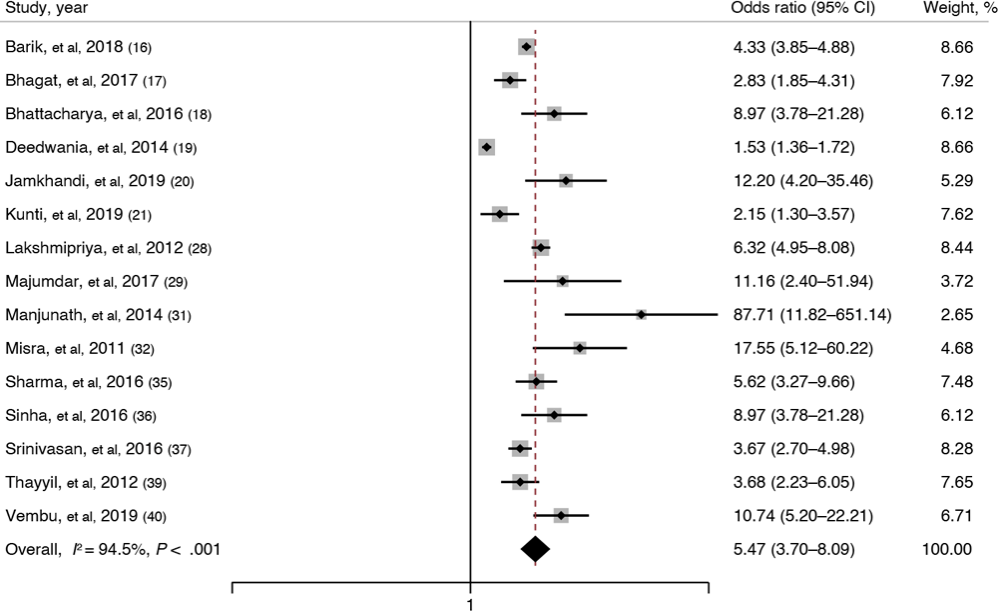
Forest plot showing the association of overweight (BMI ≥23–≥25) with metabolic syndrome among adults in India based on a systematic review of 15 studies (16–21,28,29,31,32,35–37,39,40). The definition of overweight varies among studies. Weights are from a random-effects model. The gray boxes around the point estimates indicate the preciseness of the estimate, the larger the box, the more precise the estimate (the narrower the CI).

Fifteen studies (16–21,28, 29,31,32,35–37,39,40) reported on the association between overweight and metabolic syndrome. Their pooled OR was 5.47 (95% CI, 3.70–8.09; *I* ^2^ = 94.5%; *P* < .001), indicating that overweight adults had 5.47 times higher odds of having metabolic syndrome than adults of normal and below-normal body mass index (BMI) (weight in kg/height in m^2^) (Figure 2). We also compared overweight adults with normal-weight adults only and still found a substantial association between overweight and metabolic syndrome (pooled OR, 4.49; 95% CI, 2.73–7.41; *I* ^2^ = 76%).

**Table:** 

Study, year	Odds ratio (95% CI)	Weighted %
Barik et al, 2018 (16)	4.33 (3.85–4.88)	8.66
Bhagat et al, 2017 (17)	2.83 (1.85–4.31)	7.92
Bhattacharya et al, 2016 (18)	8.97 (3.78–21.28)	6.12
Deedwania et al, 2014 (19)	1.53 (1.36–1.72)	8.66
Jamkhandi et al, 2019 (20)	12.20 (4.20–35.46)	5.29
Kunti et al, 2019 (21)	2.15 (1.30–3.57)	7.62
Lakshmipriya et al, 2012 (28)	6.32 (4.95–8.08)	8.44
Majumdar et al, 2017 (29)	11.16 (2.40–51.94)	3.72
Manjunath et al, 2014 (31)	87.71 (11.82–651.14)	2.65
Misra et al, 2011 (32)	17.55 (5.12–60.22)	4.68
Sharma et al, 2016 (35)	5.62 (3.27–9.66)	7.48
Sinha et al, 2016 (36)	8.97 (3.78–21.28)	6.12
Srinivasan et al, 2016 (37)	3.67 (2.70–4.98)	8.28
Thayyil et al, 2012 (39)	3.68 (2.23–6.05)	7.65
Vembu et al, 2019 (40)	10.74 (5.20–22.21)	6.71
Overall, *I* ^2 ^= 94.5%, *P* < .001	5.47 (3.70–8.09)	100.00

Subgroup analysis based on geographical region showed that overweight adults in the southern region had the highest estimated magnitude of association with metabolic syndrome (pooled OR, 7.06; 95% CI, 4.82–10.32). Analysis based on study setting showed that community-based studies yielded the highest estimated magnitude of association between overweight and metabolic syndrome (pooled OR, 6.24; 95% CI, 3.56–10.94). Although subgroup analysis based on the metabolic syndrome definition showed high variation in the estimated magnitude of association, that analysis was based on a single study’s estimate, not a pooled estimate. Subgroup analysis based on the quality of studies did not show any variation in the magnitude or direction of the association. Univariable meta-regression showed that none of these factors were significantly associated with the size of the pooled effect and cannot explain the substantial heterogeneity in the results.

The funnel plot showed signs of asymmetry and was also statistically proved by Egger test (*P* = .06), whereas the Doi plot showed signs of major asymmetry with an LFK index of 4.02. Sensitivity analysis showed no significant variation in the magnitude or direction of outcome, indicating a single study’s lack of influence on the overall pooled estimate. The cumulative random-effects meta-analysis also did not show any significant difference in the magnitude and direction of the pooled estimate over the range of years.

### Obesity and metabolic syndrome

**Figure 3:**
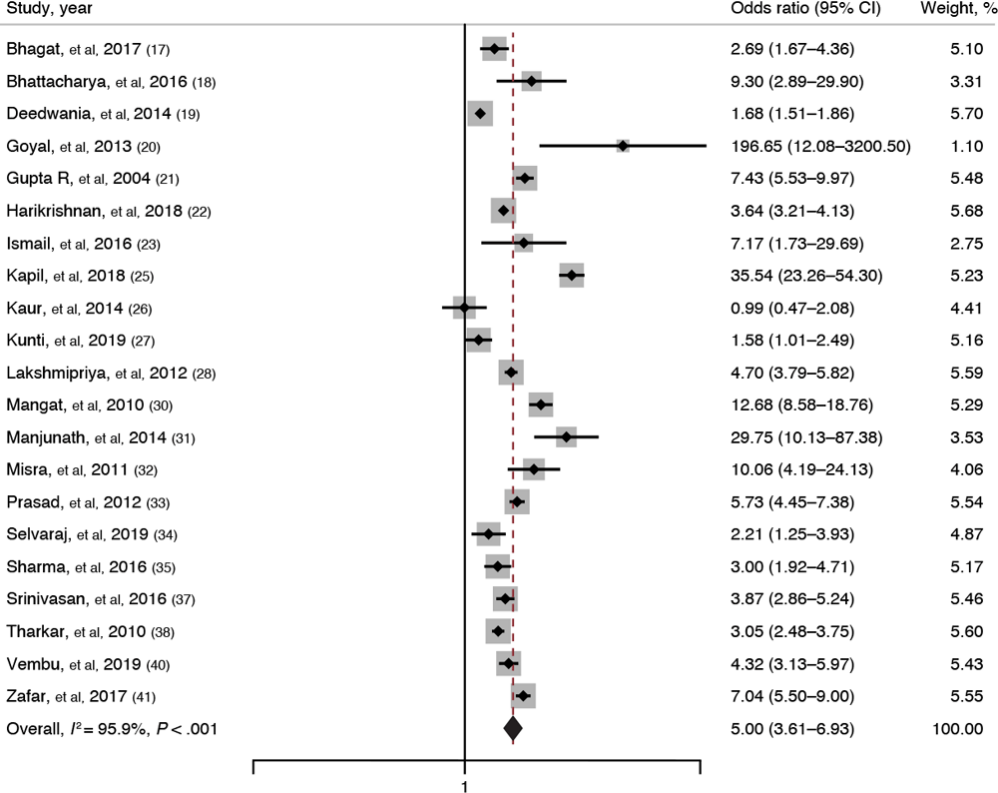
Forest plot showing the association of obesity with metabolic syndrome among adults in India, as reported in 21 studies (17–23,25–28,30–35,37,38,40,41). The definition of obesity varies among studies. Weights are from a random-effects model; continuity connection was applied to studies with zero cells. The gray boxes around the point estimates indicate the preciseness of the estimate, the larger the box, the more precise the estimate (the narrower the CI).

Twenty-one studies (17–23,25–28,30–35,37,38,40,41) reported on the association between obesity and metabolic syndrome. The pooled OR was 5.00 (95% CI, 3.61–6.93; *I* ^2^ = 95.9%; *P* < .001), indicating that obese adults have 5 times higher odds of having metabolic syndrome than nonobese adults (Figure 3). Comparison of obese adults with adults of normal BMI showed that obese adults had 4.74 times higher odds of having metabolic syndrome than those with normal BMI (pooled OR, 4.74; 95% CI, 3.19–7.04; *I* ^2^ = 90.4%).

**Table:** 

Study, year	Odds ratio (95%CI)	Weighted %
Bhagat et al, 2017 (17)	2.69 (1.67–4.36)	5.10
Bhattacharya et al, 2016 (18)	9.30 (2.89–29.90)	3.31
Deedwania et al, 2014 (19)	1.68 (1.51–1.86)	5.70
Goyal et al, 2013 (20)	196.65 (12.08–3200.50)	1.10
Gupta R et al, 2004 (21)	7.43 (5.53–9.97)	5.48
Harikrishnan et al, 2018 (22)	3.64 (3.21–4.13)	5.68
Ismail et al, 2016 (23)	7.17 (1.73–29.69)	2.75
Kapil et al, 2018 (25)	35.54 (23.26–54.30)	5.23
Kaur et al, 2014 (26)	0.99 (0.47–2.08)	4.41
Kunti et al, 2019 (27)	1.58 (1.01–2.49)	5.16
Lakshmipriya et al, 2012 (28)	4.70 (3.79–5.82)	5.59
Mangat et al, 2010 (30)	12.68 (8.58–18.76)	5.29
Manjunath et al, 2014 (31)	29.75 (10.13–87.38)	3.53
Misra et al, 2011 (32)	10.06 (4.19–24.13)	4.06
Prasad et al, 2012 (33)	5.73 (4.45–7.38)	5.54
Selvaraj et al, 2019 (34)	2.21 (1.25–3.93)	4.87
Sharma et al, 2016 (35)	3.00 (1.92–4.71)	5.17
Srinivasan et al, 2016 (37)	3.87 (2.86–5.24)	5.46
Tharkar et al, 2010 (38)	3.05 (2.48–3.75)	5.60
Vembu et al, 2019 (40)	4.32 (3.13–5.97)	5.43
Zafar et al, 2017 (41)	7.04 (5.50–9.00)	5.55
Overall, *I* ^2 ^= 95.9%, *P* < .001	5.00 (3.61–6.93)	100.00

Subgroup analysis based on the geographic region showed that obese adults in the northern region had the highest estimated magnitude of association with metabolic syndrome (pooled OR, 7.21; 95% CI, 3.33–15.61). Analysis based on study setting showed that community-based studies had the highest estimated magnitude of association between obesity and metabolic syndrome (pooled OR, 5.34; 95% CI, 3.59–7.95). Subgroup analysis based on the metabolic syndrome definition showed that studies using International Diabetes Foundation criteria reported maximum estimated magnitude of association between obesity and metabolic syndrome (pooled OR, 8.78; 95% CI, 3.87–19.91). Analysis based on the quality of studies showed no variation in the magnitude or direction of the association. Univariable meta-regression revealed that none of the factors were significantly associated with the pooled effect size and cannot explain the substantial heterogeneity in the results.

Publication bias was graphically checked by funnel plot and Doi plot. The funnel plot showed signs of asymmetry and was also statistically proved by Egger test (*P* = .03), whereas the Doi plot showed signs of major asymmetry with an LFK index of 2.96. Sensitivity analysis showed no significant variation in the magnitude or direction of outcome, indicating the lack of influence of a single study on the overall pooled estimate. Cumulative random-effects meta-analysis showed that the magnitude of this association has decreased in the past decade (2011–2020) compared with studies published in the previous decade (2001–2010).

## Discussion

The prevalence of metabolic syndrome has been increasing in India. This could further increase disease and death from noncommunicable diseases. Hence, it is important to identify patients at high risk of developing the condition as early as possible to prevent future complications. We undertook this review to study the association between anthropometric risk factors and risk of metabolic syndrome among adults in India. We found 26 studies matching the eligibility criteria for our review. Most were conducted in southern states. Though almost all the studies were cross-sectional in design, more than half were of high quality.

We found that overweight and obese adults had 5 times higher odds of having metabolic syndrome than adults with normal or low BMI. Studies around the world have reported similar findings, indicating that overweight and obese people had significantly higher prevalence of metabolic syndrome than normal or underweight people (42,43). A longitudinal study from Israel also reported that a normal BMI can rule out metabolic syndrome, which is in line with the findings from our review (44). Despite the availability of these individual studies, we found no previous reviews with which to compare our study findings. Hence, we explored possible mechanisms responsible for the association between BMI and metabolic syndrome by using the previous literature.

Much evidence has linked obesity with sympathetic overactivity (increase in heart rate, blood pressure, breathing rate) (45,46). The activation of the sympathetic nervous system has been related to insulin resistance, and weight gain leads to development of diabetes, hypertension, dyslipidemia, and several other metabolic, cardiovascular, and renal complications (47–49). Whether activation of the sympathetic nervous system and insulin resistance are the causes or consequences of obesity is uncertain (49). However, our understanding about the link between overweight and obesity and metabolic syndrome has increased with the discovery of various products released from adipocytes, such as inflammatory cytokines, leptins, non-esterified fatty acids, adiponectin, and resistin (50). In obese people, these products are released from the adipocytes in abnormal amounts and have a direct or indirect influence on the development of many metabolic risk factors (51).

In addition to studying mechanisms related to the sympathetic nervous system, we need a detailed understanding of how BMI modifies the cardiometabolic processes in humans, because the combination of obesity and metabolic syndrome has been found to be much more deadly than either disorder alone (52,53). Understanding the mechanism will help to explain the causal pathway and develop appropriate interventions. In the recent past, genome-wide association studies have been successful in identifying the large numbers of genetic loci affecting the relationship between obesity and metabolic syndrome (54). Such genome-wide association studies will be necessary over the next few years to identify and characterize the causal genes and their effects on obesity and metabolic syndrome.

The management of people who are overweight and obese with metabolic syndrome mainly focuses on lifestyle changes rather than medical management. Physical activity is an important intervention because of its role in insulin signaling, independent of the phosphatidylinositol-3-kinase pathway, and in the enhancement of glucose transporter type 4 translocation and glucose transport during the stimulation of skeletal muscles by contraction (55). This mechanism averts the risk of metabolic syndrome and was supported by the Diabetes Prevention Program study (55). Physical activity is also reported to enhance the adipose fuel metabolism because abdominal fat and fat-derived mesenchymal stem cells are more responsive to physical activity, preventing adipogenesis (56). More such evidence-based intervention planning needs to be generated through large-scale longitudinal studies and genome-wide association studies.

Analysis based on geographic region showed that adults in the northern and southern regions of India had a higher estimated magnitude of association between obesity and metabolic syndrome than other regions. A possible reason for this finding could be the higher magnitude of risk factors for noncommunicable disease among adults residing in northern and southern states compared with that of other regions. The latest report of India’s National Nutritional Monitoring Bureau showed that the northern and southern states have a higher degree of risk factors, such as poor dietary habits, diabetes, hypertension, and hypercholesterolemia, than other regions (57). Social determinants of health also might have influenced the higher magnitude of association in these regions. A nationwide study assessed the progress and inequities in the social determinants of health (eg, education, child mortality and underweight, water, sanitation, fuel use, housing, electricity) across the geographic regions of India on the basis of multiple nationwide household surveys (58). That study reported that many states in the northern region of the country are more socioeconomically disadvantaged, a condition that increased with each round of the surveys over the past 2 decades. Hence, the possible confounding effect from both medical and nonmedical factors could be responsible for the uneven magnitude of association between BMI and metabolic syndrome across different regions.

The major strength of our review was the rigorous literature search and methodology followed to provide reliable estimates. In addition, this was the first review providing the association between anthropometric risk factors and metabolic syndrome among adults in India. We also found that most of the 26 studies included in our review were of high quality and had standard criteria for defining overweight and obesity, which might make our study findings generalizable. We also found no significant change in the magnitude and direction of the association between metabolic syndrome and BMI with respect to the exposure–outcome relationship in sensitivity analysis.

Our study also had limitations. Our results should be interpreted with caution and inferred accordingly, considering the difference in methods and quality across the included studies. In our analysis, we found significant between-study variability (χ^2^ test for heterogeneity and *I* ^2^ statistics were significant). We explored the reason for such high heterogeneity by using meta-regression analysis. However, we found no factors attributable to the differences among the included studies. In addition, we found the possibility of publication bias with both overweight and obesity, limiting the credibility of the generated evidence. Most of our included studies were cross-sectional in nature, which made it difficult to establish causation between the exposure and disease. Hence, more longitudinal studies are needed in India to identify accurate and reliable effect estimates and make evidence-based recommendations for reducing the BMI level among the general population.

We found overweight and obesity to be significantly associated with metabolic syndrome. Given the evidence, clinicians and policy makers alike should implement weight reduction strategies among their patients and the general population. Though our results provide some crucial information for a better understanding of the association of anthropometric risk factors and metabolic syndrome, longitudinal studies are still needed to establish the temporality of the association and the causal link. Understanding this causal link with special focus on the dose–response relationship will overcome a crucial barrier in the management of patients with metabolic syndrome and help prevent many cardiovascular diseases and deaths worldwide.
